# A New Approach to Evaluating Aberrant DNA Methylation Profiles in Hepatocellular Carcinoma as Potential Biomarkers

**DOI:** 10.1038/srep46533

**Published:** 2017-04-18

**Authors:** Yuan Yang, Linghao Zhao, Bo Huang, Guojun Hou, Beibei Zhou, Jin Qian, Shengxian Yuan, Huasheng Xiao, Minghui Li, Weiping Zhou

**Affiliations:** 1 The Third Department of Hepatic Surgery, Eastern Hepatobiliary Surgery Hospital, Second Military Medical University, Shanghai, China; 2 Suzhou Municipal Hospital, Jiangsu Province, China; 3 Shanghai Biotechnology Corporation, Shanghai, China

## Abstract

Hypermethylation of CpG islands in the promoter region of tumor suppressor genes (TSGs) and their subsequent silencing is thought to be one of the main mechanisms of carcinogenesis. MBD2b enrichment coupled with a NimbleGen array was applied to examine the genome-wide CpG island methylation profile of hepatocellular carcinoma (HCC). Hypermethylated DNA of 58 pairs of HCC and adjacent tissue samples was enriched and hybridized in the same array. Aberrant hypermethylated peaks of HCC and adjacent tissues were screened and annotated after data processing using NimbleScan2.5 and our newly developed Weighting and Scoring (WAS) method, respectively. Validation using bisulfite sequencing of randomly selected *ANKRD45, APC, CDX1, HOXD3, PTGER* and *TUBB6* genes demonstrated significant hypermethylation modification in HCC samples, consistent with the array data.

Cytosine methylation is the most common epigenetic modifications of DNA and occurs at CpG dinucleotides to form the CpG islands’ structure^1^,^2^,^3^. CpG islands are among the most important regulatory elements in the human genome^4^,^5^. As the most intensely studied epigenetic modification, various functions of DNA methylation have been discovered, such as gene expression regulation, gene imprinting, X-chromosome inactivation, maintenance of chromatin stability, and cancer-associated regulation^6^,^7^.

Many studies have reported aberrant methylation patterns of both genome-wide hypomethylation and gene-specific hypermethylation in cancer^8^,^9^,^10^,^11^. For example, dozens of cancer-related genes have been found to be both hypermethylated and expression-silenced in cancer. Thus, numerous studies have been devoted to discovering the mechanisms of tumorigenesis and to developing diagnostic methods using methylation biomarkers^12^,^13^.

During the last decade, various techniques have been developed to study the mechanisms of methylation-related DNA modification, and most of them involve two steps: identification (or enrichment) and detection. The direct identification of individual methyl groups is a difficult task. However, substitutions, bisulfite modification, methylation-sensitive endonucleases, and methylation affinity chromatography by MBD proteins or antibodies have been widely used in methylation identification. The MBD proteins such as MBD1, MBD2, MBD3, MBD4 and MeCP2 belong to a family of nuclear proteins with a methyl-CpG binding domain (MBD), which has specific affinity to methylated DNA^14^,^15^. In mammals, the MBD3 binds 5-hydroxymethylcytosine, and the MeCP2, MBD1, and MBD2 are the major methylated DNA-binding proteins *in vivo* and down regulation the gene expression^16^,^17^. The MBD2b is a shorter variant of methyl-CpG-binding domain protein-2 (MBD2) lacking the N-terminal 140 amino acids but with fully methyl-CpG binding domain^18^. Exprimets *in vitro* shows that recombinant the mouse MBD2b protein have the highest affinity to metylated DNA among mouse MeCP2, MBD2b and MBD3, and Xenopus MeCP2, MBD3 and MBD3 LF^19^. MBD2b is the widely choosed protein for enrichment of metylated DNA in many studies and can be coupled with MBD3L1 to gain higher affinity to methylated DNA^20^,^21^,^22^,^23^.

Three technologies, electrophoresis, sequencing and microarray, can be used to identify the level of methylation. The development of these detection techniques has progressed from a single locus or gene (COBRA, BSP, MSP) to a whole-genome assay (MIRA, MeDIP), and then to high-throughput methods such as next-generation sequencing (NGS) and whole-genome array^24^,^25^,^26^. The ChIP-chip method is one of the most widely used techniques to identify genome-scale methylation profiles. All these methods are based on affinity, and bisulfite treatment can produce accurate DNA methylation data^27^. However, it is more convenient and economical to analyze dozens of samples by array-based methods rather than NGS because the array data are smaller than those from NGS and are easily processed because they are in the same format. ChIP on chip data cannot provide single base resolution; however, its 50-bp resolution is adequate for DMR (Differentially Methylated Region) screening in hepatocellular carcinoma (HCC).

HCC is one of the most common malignancies and the third cause of death in males in the world. In this study, we selected 58 pairs of HCC and adjacent liver tissue samples to analyze the genome-wide methylation level. Glutathione S-transferase (GST)-tagged recombinant MBD2b protein was used to identify or enrich the methylated DNA fragments, which were then hybridized to a DNA methylation array (NimbleGen 385 K human whole-genome CpG island chip, NimbleGen, Inc., Madison, WI, USA)^28^. Here, a new computational strategy, the Weighting and Scoring (WAS) method, was developed to evaluate the relative methylation level of each predicted CpG island in HCC. Additionally, some new epigenetic biomarkers were identified that exhibited potential for the early detection of HCC.

## Results

### Quality and efficacy of the enrichment

**Figure 1:**
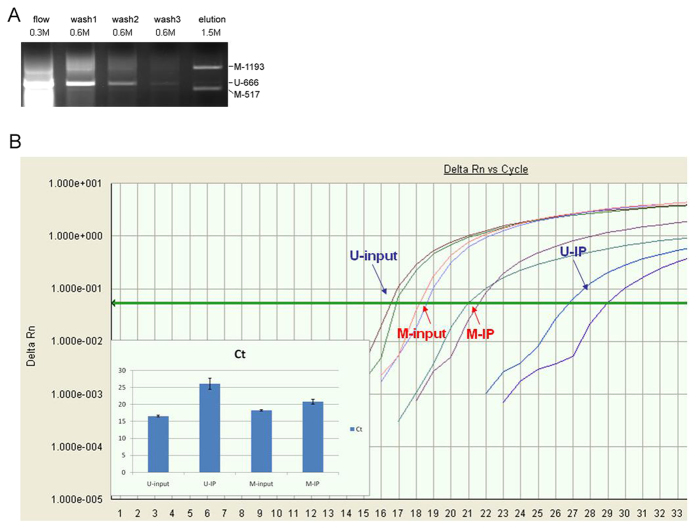
GST-MBD2b enrichment quality control and efficacy. (**A**) Two methylated DNA fragments (1193, 517) and one unmethylated fragment (666) were incubated with MBD2b resin to test the binding affinity of MBD2b to the methylated DNA fragment. Although the unmethylated fragment band signal diminished after washing, the methylated fragments remained and could be recovered in the elution fraction. (**B**) Quantitation of positive and negative control fragments in the enriched sample. Abbreviations: U-input: unmethylated primer test in the input sample; U-IP: unmethylated primer test in the IP sample; M-input: methylated primer test in the input sample; M-IP: methylated primer test in the IP sample. The increase in the Ct value with the U primer was greater than that of the M fragment indicating that the recovery ratio of the M fragment was greater than that of the U fragment.

The affinity of GST-MBD2b to methylated DNA fragments was tested using two methylated DNA fragments and an unmethylated fragment as a control. The sample wash through, wash and elution fractions were electrophoresed on a 1% agarose gel. Successful enrichment was defined as the detection of methylated fragments only in the elution fraction (Fig. 1A).

We mixed two PCR fragments amplified from an *Arabidopsis* CpG island region with the genomic samples. As a positive control, one fragment was treated with SssI, and the other served as a negative control. The enriched samples were analyzed by quantitative PCR, and the ratio was calculated after the binding procedure. For all 58 pairs of samples, each enrichment ratio of positive/negative control ranged from 33- to 200-fold (Fig. 1B). We chose the samples in which the ΔΔCt was greater than 5. However, the copy number of the unmethylated fragments after enrichment will decrease to a minimum, the Ct value in quantitative PCR will approach 40, and the Ct value may fluctuate and result in overestimation of the enrichment rate.

### Evaluation of CpG island methylation status in HCC

**Figure 2:**
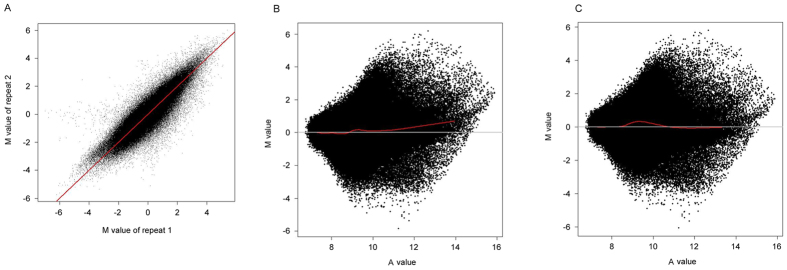
Correlation of microarray replicates and the print-Tip Loess normalization effect. (**A**) Correlation of microarray replicates. (**B**), (**C**) Correlation after data normalization.

The signal for each probe in our microarray was calculated after background subtraction. The correlation coefficients of microarray replicates were greater than 0.9 (Fig. 2A). After data normalization (Fig. 2B,C), the log_2_ ratio of the Cy3/Cy5 signal for each probe was calculated.

A total of 27,353 predicted CpG islands were identified in our microarray. We selected 20,779 CpG islands, which contained 4~20 probes, for subsequent analysis using the WAS method.

**Figure 3:**
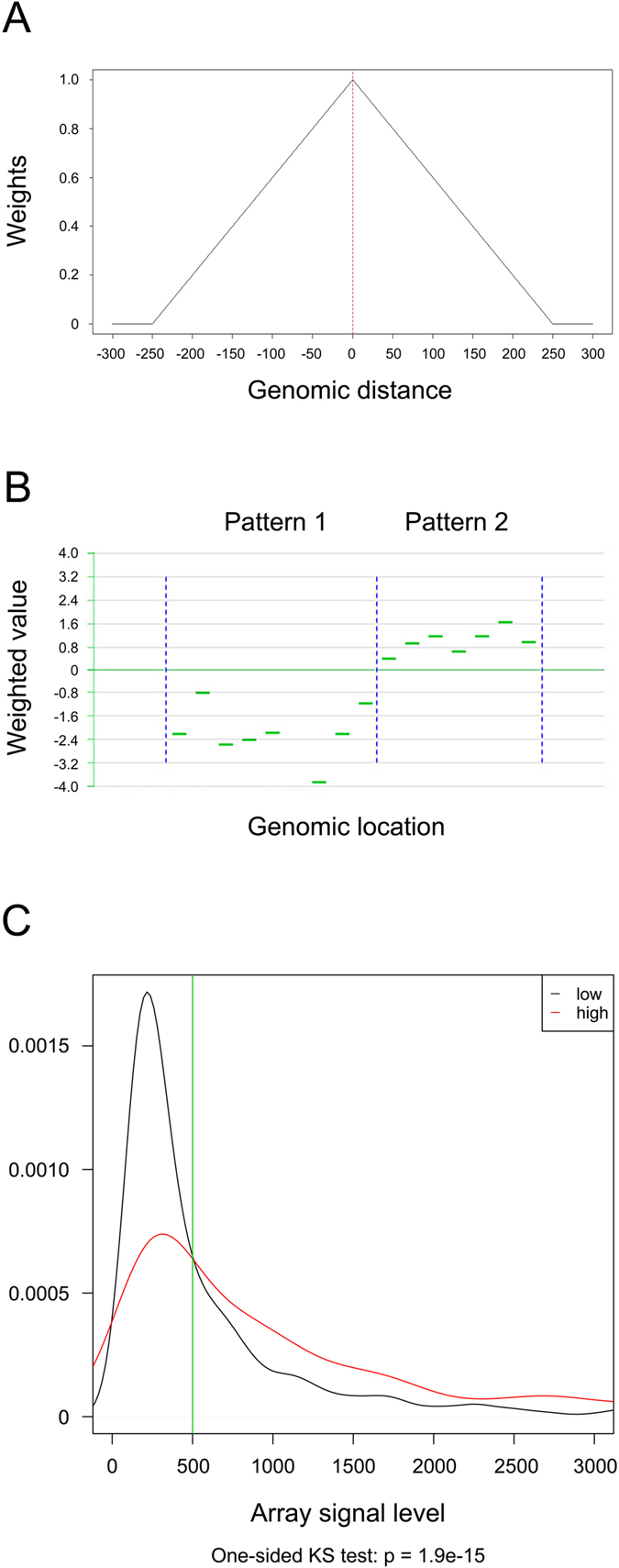
(**A**) Weighted value of the probe processed: all probes within the range of 500 bp were calculated. The weight of a probe depends on its distance from the center of the range. (**B**) Pattern recognition. (**C**) Signal cutoff for CpG islands.

**Figure 4:**
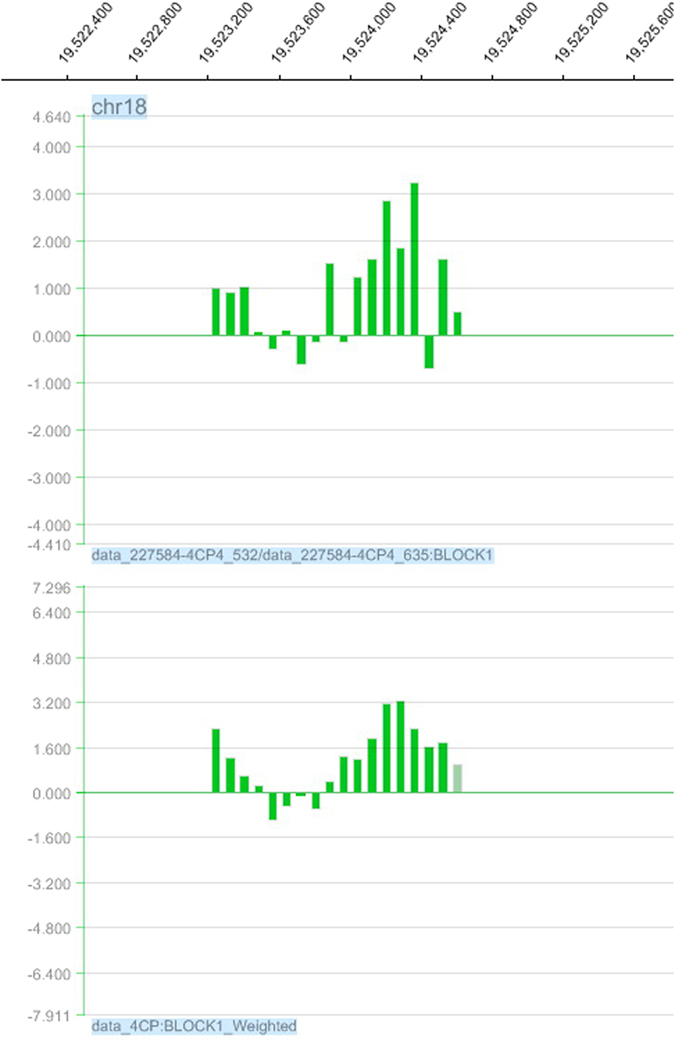
Example of the probe log_2_ ratio before and after data processing using WAS in signalmap view.

Considering the characteristics of our microarray design, the signal of each probe can be influenced by neighboring probes, referred to as the “neighbor effect”^29^. This effect can be represented by a “weighting” procedure (Fig. 3A). The log_2_ ratio values were transformed to a weighted value according to the distance to other adjacent probes (Fig. 3A). The effect after probe weighting is shown in Fig. 4.

After probe weighting, each CpG island was sorted into several patterns (Fig. 3B), in which the probe weighted values had the same signs. The significance of each pattern was analyzed by t-test (p < 0.05). Significant patterns were selected, and the significance of the probes was analyzed by t-test (p < 0.05). After excluding the insignificant probes, a score for each selected pattern was obtained (see Methods). If one CpG island had only one significant pattern, then its final score corresponded to the pattern score; if not, then the score was equal to the mean score of all corresponding patterns.

### Signal cutoff for filtering undetected CpG islands

The signal level of probes from hypermethylated CpG islands could be significantly distinguished from those of probes from hypomethylated CpG islands by BSP validation (Fig. 3C; one-sided Kolmogorov-Smirnov test: p = 1.9 × 10^−15^). The junction of these two density curves was at approximately 500 (Fig. 3C, the vertical line). If the signal levels were less than this value, the validation error rate (or probability) would be much increased because of the inadequately low signal level. Thus, we could consider that probes with a signal less than 500 were “undetected”, and those CpG islands with more than 80% of undetected probes in both channels were assigned “N/D” (not detectable) scores.

### Hypermethylated genes in HCC

**Figure 5:**
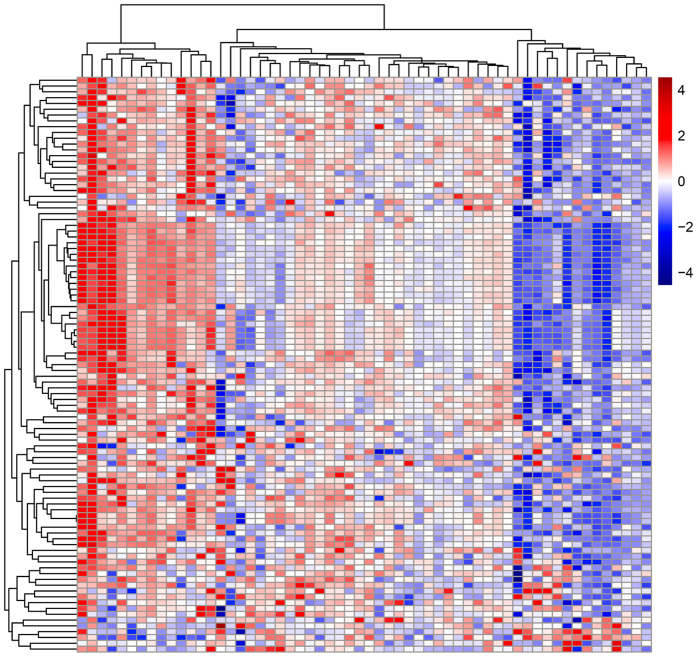
A heatmap showing the cluster pattern of the top 100 sites, with significant hypermethylated and hypomethylated genes and CpG loci in 58 pairs of HCC and adjacent tissues, using a hierarchical clustering algorithm. Red indicates hypermethylation, whereas blue indicates hypomethylation.

We developed the WAS method to determine the difference between HCC tissues and the corresponding adjacent tissues. If one CpG island site in a pair of tissues was significantly different, then the peak of this CpG island site could be found. We found 317 consistent peaks in more than 30 samples, 552 peaks in 20 to 29 samples, and 493 peaks in 10–19 samples. Figure 5 demonstrated the top 100 hypermethylated and hypomethylated sites in HCC ones. To study the association between these genes and the reported TSGs (tumor suppressor genes), we selected 66 reported TSGs (or related genes) (Supplementary Table 2) to analyze their distribution in our hypermethylated sites. Among these 66 genes, 32 were found in our results, and 13 of them (Table 1) had hypermethylated peaks in more than 10 samples. The methylation of the *PGR, MYC* and *APC* genes was most highly correlated with HCC (in 21–23 samples). Furthermore, some TSGs have more than one transcriptional start site or CpG island, such that different peaks may be found in one gene, such as in the CDKN1B and *APC* genes. The *CDH15, CDH1, MYC*, and *CDKN2B* genes showed hypermethylated and hypomethylated peaks in HCC, and the variation of the methylation state in these genes indicated that methylation silencing might play important roles in carcinogenesis.

In our hypermethylated gene list (Table 1), *CDKN2A, CDKN1B*, and *APC* have been reported to be hypermethylated in many types of tumors, including HCC^30^,^31^,^32^. Some genes, such as *DAPK3, PRLR, PYCARD* and *MGMT*, were hypermethylated in only 2 or 3 samples. In addition to these known TSGs, some imprinting genes (e.g., *PEG3, SNRPN, KLF14, KCNQ1DN* and *ATP10A*) were more commonly hypermethylated. *H19, NDN, DLK1, DIRAS3* and others are hypomethylated in HCC tissue, which indicated the gain of imprinting in HCC tumors and the loss of heterozygosity of imprinting genes in tumors. Therefore, these genes may be valuable for diagnostic purposes.

### Bisulfite PCR sequencing validation

**Figure 6:**
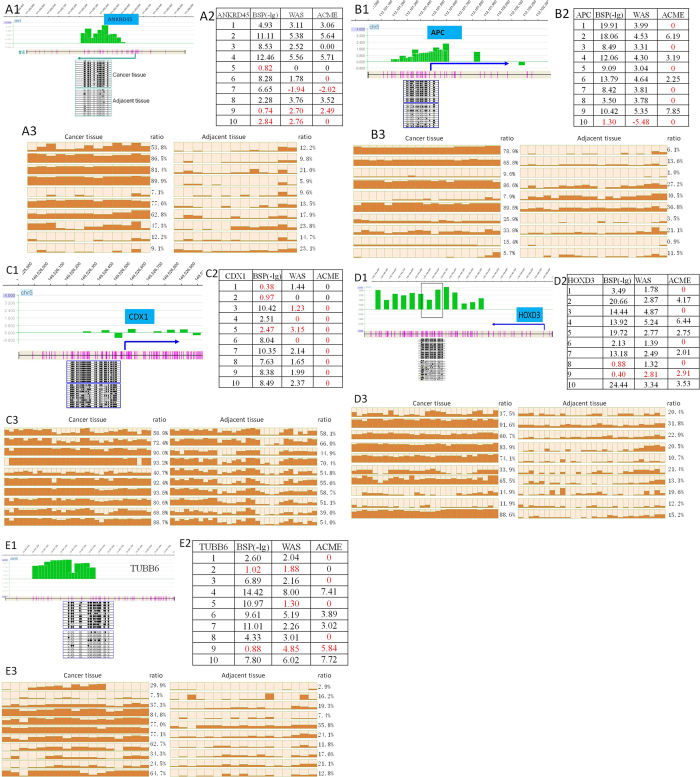
Examples of WAS scores and corresponding BSP results, with CG dinucleotide location and probe weighted values for randomly selected genes. (**A**) *ANKRD45*, (**B**) *APC*, (**C**) *CDX1*, (**D**) *HOXD3* and (**E**) TUBB6. Panel 1. Chromosomal position of the probe, CG site, gene and BSP clone sequencing result for each gene. Green histograms show the position and log ratio data of each probe. Purple line indicates the CG site position of the CpG island, and the arrow represents the gene transcript start site and orientation. BSP clone sequencing results for one pair of HCC and adjacent samples are shown in the corresponding position. Panel 2. Table of -log_10_ p values of BSP validation results, WAS score and -log^10^ p value of ACME results for 5 genes. The first columns denote the sample ID. The second columns denote the t-test p values for methylation greater in HCC than adjacent tissue in all 10 samples. If HCC methylation is significantly less than that in the adjacent tissue, then the p value should be greater than 0.95. The third columns denote the WAS scores, and the last columns denote the ACME p values. Red color denotes incorrect results. Yellow and gray colors denote false positive and false negative results, respectively. Panel 3. BSP clone sequencing result of 10 pairs of tumor (left) and adjacent tissue (right), histogram of the methylation ratio of each CG site, and the total ratio of methylation are also listed.

Hundreds of CpG island segments were found to be consistently hypermethylated or hypomethylated in HCC in more than 2/3 of the samples by our WAS method. The islands near the promoter regions were candidates for validation to screen for potential diagnostic biomarker(s). Sodium bisulfite sequencing (BSP) was used to validate the 6 randomly selected candidate islands, in which the results of 5 mapped genes (*ANKRD45, CDX1, APC, HOXD3* and *TUBB6*) showed significant differences in the 10 pairs of validation tumor and adjacent tissue cohorts. We counted the methylated CG dinucleotides and unmethylated ones and tested the significant difference between them (Fig. 6A). For example, in the *ANKRD45* gene, 7 samples had significantly different hypermethylation by BSP in a total of 10 samples (t-test, p < 0.05). Among the samples, 7 were correctly scored by our WAS method, and 6 were correctly identified by ACME, another analytical method^29^ (see Discussion). Other genes also showed the correct ratio of approximately 2/3 (not including the false negative results) for the WAS method compared to BSP validation. Similar results were found in the *APC* gene (Fig. 6B). In the *CDX1* gene, the WAS method identified a slight difference between the tumor and adjacent tissue in 4 samples, but ACME detected no differences (Fig. 6C).

**Figure 7:**
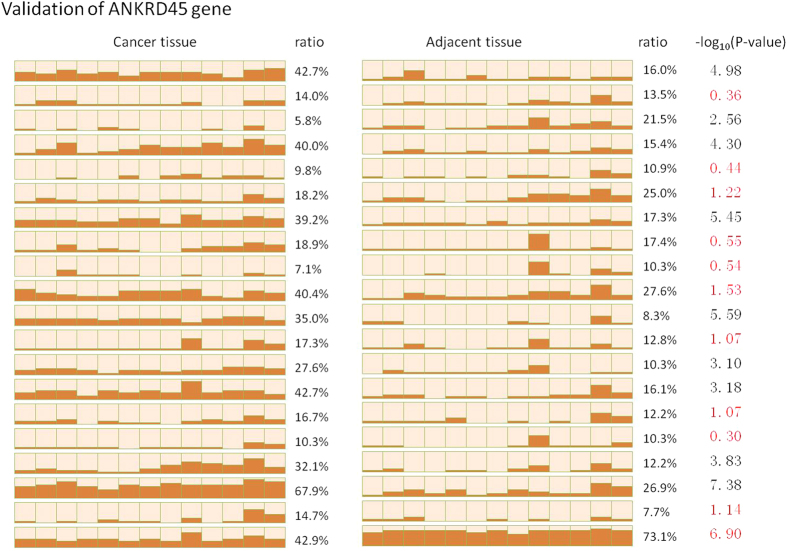
Validation of the *ANKRD45* gene in 20 more HCC tissue and adjacent tissue samples.

Some BSP results for the *ANKRD45* gene are shown in Fig. 6. Significantly hypermethylated peaks of the CpG island and a positive correlation of the WAS score with BSP sequencing results were found in sample R4, and similar results were also found in samples R1, R2, R3, R6 and R8. We studied the methylation pattern of *ANKRD45* for another 20 pairs of HCC and adjacent tissue by BSP cloning sequencing and found that 9 (45%) of them showed significant hypermethylation in HCC (Fig. 7).

### Expression of the *ANKRD45* and *HOXD3* genes

**Figure 8:**
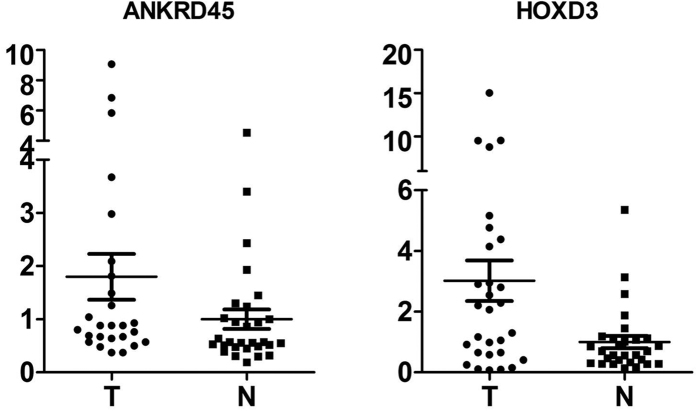
RT-PCR result of the expression level of the *ANKRD45* and *HOXD3* genes in HCC tissue and adjacent tissue.

We studied the expression level of the *ANKRD45* and *HOXD3* genes in cancer tissue and adjacent tissue using quantitative PCR. We found that the expression level of these two genes in cancer tissue was higher than in the adjacent tissue (Fig. 8). The difference in expression of the *ANKRD45* and *HOXD3* genes was significant, with p values of 0.05 for *ANKRD45* and 0.005 for *HOXD3*.

## Discussion

### MBD2b enrichment method

This study shows the high throughput and specific experimental platform of immunoprecipitation-coupled whole genome CpG island chip. We used GST-tagged MBD2b combined with Sepharose 4b chromatography to enrich the methylated DNA from sonicated genomic DNA rather than digestion, presenting an unbiased profile of whole-genome methylation. The methyl-CpG binding proteins may offer a great advantage over methylation-sensitive restriction enzymes because the enzymes may only recognize a part of the methylated sites in the genome, even when 5 enzymes are used together^33^. We chose the MBD2b protein for enrichment because it has been reported to have the highest affinity to hypermethylated DNA among the members of the MBD family^19^. The BMD2b protein prefers to bind to the hypermethylated DNA than the antibody, which specifically binds to the 5 mC in single-stranded DNA (ssDNA), giving a more representative result. However, another report has shown a similar result with the MeDIP and MethylCap methods with His6-GST-MBD^27^. Methylated DNA enrichment is a technique under development to evaluate the methylation status and may be affected by the frequency of CpG dinucleotides in particular sequences. The method of whole-genome bisulfite sequencing by high-throughput sequencing technology will likely be a promising technique^25^,^26^, but its high cost makes ChIP-chip a more economical method for a whole-genome methylation study.

### Effect of different enrichment rates

We used quantitative PCR to evaluate the efficacy of enrichment; samples with insufficient or excessive enrichment were discarded to obtain consistent results. However, it is impossible to achieve the same ΔΔCt value in different samples, which are influenced not only by the difficult operation of ChIP but also by errors in quantitative PCR. Thus, we selected samples with a ΔΔCt value between 5 and 8, which indicates 32- to 256-fold differences between fully methylated and unmethylated fragments. The variation of the ΔΔCt is mainly due to the trace amount of unmethylated fragments in the enrichment, whose Ct approaches 35 and is not steady. These different enrichment rates will result in bias in the array results, which is mainly due to weak signals from unmethylated fragments. However, the signal cutoff will exclude these data, and the normalization procedure will reduce this effect inside the array and between arrays.

### CpG island array hybridization method

We used a custom-designed human CpG island array to analyze the differential methylation profile of HCC and adjacent tissues. This array includes a total of 27,353 islands covering ~1% of the human genome, providing higher resolution for each CpG region and a less expensive alternative to a whole-genome tiling array. However, the CpG island array has limitations. The probes are designed for a subsection of the genome, so this array may produce bias compared to hybridizing the IP sample and input sample to one chip because an equal amount of samples yields very different signals in this type of chip. The IP sample may be enriched for hypermethylated fragments that are distributed mostly in the CpG island region and thus presents a hybridization signal. However, the input sample has an average distribution in the whole genome, and only a small portion can form a signal in the array. The disequilibrium of the total amount of signal is normalized, and the input signal increases substantially before the log-ratio data processing. Thus, the normalization procedure may hide a great number of the methylated peaks by increasing the input signal, which makes estimating the degree of DNA methylation difficult. A whole-genome tiling array may avoid this bias because its probes cover the entire genome and the enriched peaks will be more prominent than others as a result. We hybridized two enriched samples, both of which preferred the CpG island region, in one array to avoid this bias. These two samples were tumor tissue and adjacent tissue from the same patient to avoid individual differences. This method may enable better evaluation of the differences between the tumors, or between the tumor and adjacent tissue in one array.

The method of hybridizing tumor and adjacent tissue in one array also has its limitations. False positive results may arise in areas of low signal level, where the DNA is hypomethylated and little difference can be found between the tumor and adjacent tissues. Further validation methods are needed to determine the hypermethylated sites in tumors.

An antibody may not improve the result; however, the antibody affinity increases with the number of methylated CpG sites from approximately 1 to 12, and it was difficult to distinguish differences in higher numbers of methylated CpG sites^34^.

### Hypermethylated genes in tumors

We selected hypermethylated genes rather than hypomethylated genes in HCC for validation by BSP because they may be candidate TSGs. Thus, validating these data would be helpful to determine TSGs and biomarkers for HCC diagnosis.

Some of these TSGs, e.g., *MLH1, SFRP4, CDKN2A, ZMYND10, APC, GSTP1* and *PRDM2*, and some imprinting genes, such as *PEG3, SNRPN, KLF14, ATP10, H19, NDN, DLK1* and *DIRAS3*, were hypermethylated or hypomethylated in HCC tissue compared to adjacent tissue. The gain of imprinting genes in HCC and the loss of heterozygosity of the imprinting genes in tumors have been reported, and these genes may be promising for cancer diagnosis.

We also selected the peaks found by ACME, and the following genes had hypermethylated peaks in more than 3 samples: *MLH1, SFRP2, VHL, APC, FHIT, GATA6* and *RARB. MLH1* had hypermethylated peaks in 7 samples, and *SFRP2*, VHL had peaks in 5 samples, which were highly correlated with HCC. However, these samples may have peaks in different locations of the same gene.

However, some genes have more than two or more transcript start sites and CpG islands. Therefore, multiple peak sites may be found in these genes (such as *CDKN2A*).

### Comparison with the ACME method

We only selected CpG islands with fewer than 20 probes because CpG islands with too many probes may have patterns too complex and too difficult to evaluate by a single score.

As a well-tested and general purpose normalization method, print-tip Loess has yielded good results in a large number of microarrays^35^,^36^. Thus, we adopted this method to normalize our array data. The system excursion between Cy3 and Cy5 could be effectively eliminated after normalization (Fig. 2).

We have noted previously described methods for the analysis of a whole-genome tiled array, such as ACME (Algorithm for Capturing Microarray)^29^. The ACME method was developed to detect peaks in a tiled array for ChIP-chip experiments, using whole genome DNA as a reference. And the ACME algorithm is the formal method to process the Nimblegen array, that was embedded in the official software of Nimblegen scan. The ACME identifies “peaks” in tiled array data using a simple sliding window and assigns a p value to each probe on the array. ACME performs well with two different populations of labeled DNA (ChIP- or DNase-enriched DNA/total genomic DNA). The large difference between immunoprecipitated (IP) samples and input samples makes it easy to detect peaks of the methylated fragments relative to the genomic background. Thus we choose ACME method to compare with WAS method. However, the tumor and adjacent tissue have a similar methylation mode, and small differences between them can be identified by hybridizing them to one array rather than inter-array. ACME can identify the difference between enriched DNA and total genomic DNA but may not be suitable for detecting small differences between two samples with a similar methylation mode. For example, the *CDX1* gene, with hypermethylation detected in both tumor and adjacent tissues, had 31 CG dinucleotides in a 326 bp fragment, which was 53–94% methylated in most samples. This result could be because the MBD2b protein prefers to bind to this fragment^19^. However, small differences were detected in our WAS method but could not be identified by the ACME method (Fig. 6C2). The WAS method could be more sensitive and provide more useful information for the CpG island array. The WAS method was more consistent by BSP validation than the ACME method for all 5 genes in 10 pairs of samples, not considering the accuracy rate of the array data. The consistency ratio of WAS is 76% (36 in 50) and that for ACME is 44% (22 in 50).

### The expression of the *ANKRD45* and *HOXD3* genes

We analyzed the expression level of the aberrantly methylated *ANKRD45* and *HOXD3* genes. However, the expression level in HCC was higher than that in adjacent tissue, and hypermethylated DMR was detected in the promoter region of these two genes. The association of up-regulation of expression by methylation has been reported in the *ITPKA* gene in many forms of cancer^37^. As aberrant methylation and gene expression were observed in HCC cancer and adjacent tissue, we hypothesize that the expression may be upregulated by the modification of methylation.

## Materials and Methods

### Sample preparation

Frozen HCC tissues and adjacent tissue of 58 Chinese patients were prepared for this study. The demographic and clinical characteristics of the patients were systematically collected and are summarized in Supplementary Table 3. The inclusion criteria of this study were as follows: (i) HBV-positive HCC and paired adjacent non-tumor tissues, (ii) tissues obtained from consenting patients, (iii) all samples are HCV-negative and HIV-negative, and (iv) without autoimmune hepatitis and metabolic and/or genetic disorders such as Wilson’s disease and hemochromatosis. DNA was extracted from 30 mg of tissue by phenol extraction and ethanol precipitation.

### Ethics statement

This study was approved by the Ethics Committee of Eastern Hepatobiliary Surgery Hospital, and informed consent was obtained from each patient.

### Methylated CpG island enrichment

The full-length *MBD2b* gene was cloned by reverse transcription PCR using forward primer: 5′-GCGTCAGGGATCCCCATGCGCGCGCACCCGG-3′, and reverse primer: 5′-GCGTCTGCTCGAGTGGAGGAAAGGATTGGTT-3′. PCR products were cloned into the pGEX-5X-1 expression vector (Amersham Pharmacia Biotech, Piscataway, NJ) and transduced into BL21 to express glutathione-S-transferase (GST)-tagged recombinant MBD2b protein, which was then purified by Sepharose 4b chromatography. The inserted fragment was confirmed by sequencing.

Genomic DNA was sonicated to the size range of 300–1000 bp, ligated with adaptors (Jw102, 5′-GCGGTGACCCGGGAGATCTGAATTC-3′; Jw103, 5′-GAATTCAGATC-3′), and then enriched using MBD2b protein with the procedure adapted from the MIRA method (1). Briefly, 50 μl of Sepharose 4b (Amersham Biosciences) saturated with GST-tagged MBD2b was incubated in 200 μl of binding buffer [25 mM HEPES (KOH) (pH 7.5), 300 mM KCl, 12.5 mM MgCl_2_, 10% glycerol (sterile), 1 mM DTT]. Linker-ligated DNA (500 ng) was added to this mixture that was then incubated for 2 hours at 4 °C on a rotating platform. Sepharose beads were washed three times with washing buffer [25 mM HEPES (KOH) (pH 7.5), 600 mM KCl, 12.5 mM MgCl_2_, 10% glycerol (sterile), 1 mM DTT] and eluted with elution buffer [25 mM HEPES (KOH) (pH 7.5), 1.5 M KCl, 12.5 mM MgCl_2_, 10% glycerol (sterile), 1 mM DTT]. After elution, enriched methylated DNA was purified using QiaQuick PCR purification kits (Qiagen, Valencia, CA) and then amplified using primer Jw102 (5 μl) in 24 cycles of amplification (94 °C for 25 s, 60 °C for 25 s, and 72 °C for 90 s).

### Enrichment efficacy evaluation

After the GST-MBD2b protein was expressed using the BL21 bacterial strain, quality control using two different methylated PCR fragments was performed to guarantee affinity to the chromatography column. Primer pairs (Supplementary Table 1) were used to amplify three fragments in the *Arabidopsis* genome, and the first two were digested with SssI to be used as methylated fragments. Each fragment (500 ng) was subjected to GST-MBD2b chromatography following the above method. The enriched product was observed on a 1% agarose gel.

A one-tenth aliquot of the enriched product of the cancer tissue and adjacent tissue was diluted and tested by real-time PCR (ABI7300) to validate the efficacy of the enrichment. Methylation RT primers (Supplementary Table 1) were used to detect the recovery ratio of the methylated *Arabidopsis* fragments, and the unmethylation RT primers were used to detect the unmethylated fragments. The enrichment was considered acceptable according to the 2 following rules: the ratio of the methylated fragment to unmethylated fragment was more than 20-fold after the enrichment calculated by the 2^−ΔΔCt^ method^38^ and for each pair of HCC and adjacent samples, the ratio value should be approximately the same.

### CpG island DNA array design and hybridization

A human whole-genome CpG island array was designed. A CpG island was defined as GC% > 57% and length >250 bp; those with a repeated sequence were removed. A total of 339,175 probes were designed for 27,353 selected CpG islands, in which the probe length was 50 bp and the gap was approximately 30 bp. Another 37,452 probes were designed corresponding to 66 imprinting genes, 96 housekeeping genes, and 1,274 cancer- or cell cycle-related genes.

After elution, enriched methylated DNA was purified using QiaQuick PCR purification kits (Qiagen, Valencia, CA) and then amplified using primer Jw102 (5 μl) in 24 cycles of amplification (94 °C for 25 s, 60 °C for 25 s, and 72 °C for 90 s). The fragments amplified from the HCC and the adjacent tissues were labeled using Cy3 and Cy5, respectively, and were mixed to hybridize on one chip following the NimbleGen protocol.

### Microarray data analysis by WAS method

#### Microarray data pre-processing

R packages (limma and marray) were used for the pre-processing procedures for DNA methylation microarray data, including data read-in and normalization. The print-Tip Loess method was used for normalization. After normalization, each probe had a log_2_ ratio that was calculated as follows:


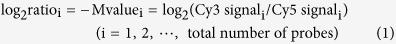


#### Weighting the probes

For each CpG island, the log_2_ ratio for probe i was transformed to a weighted value w_Probe_i_,


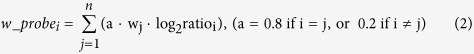


where n denotes the probe amount in each CpG island in which probe i is located, and


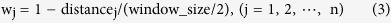


where distance_j_ denotes the nucleotide distance of probe j from probe i, and window_size denotes the size of the DNA fragments hybridized. However, those CpG islands with at least 80% of probes whose signals were less than 500 were excluded and were not weighted and scored.

#### Scoring the CpG islands

All of the weighted values were sorted by genomic order. In those CpG islands where these significant probes were included, the patterns of neighboring probes with continuous positive or negative signs were identified. A Wilcoxon rank test was then used to analyze the significance of each pattern (p < 0.05), and the probes with a value approximating 0 were filtered. A score was defined to denote the combined methylation degree of each pattern:


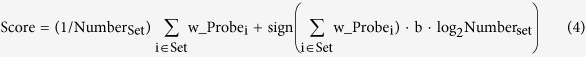


where Set denotes the probe set of each pattern excluding those filtered probes, and b is a modification factor to the score. Here, we set b = 0.5 after careful estimation.

After pattern scoring, each CpG island was assigned an average score of its patterns. Here, we used >1.5 as the cut-off value to define a hypermethylated CpG island.

The top 100 sites exhibiting different methylation scores of the CpG islands, i.e., consistently hypermethylated or consistently hypomethylated, were screened and clustered using a hierarchical clustering algorithm.

### Mapping CpG island sites to genes

NimbleScan 2.5 (NimbleGen, Inc., Madison, WI, USA) was used to map CpG island sites to genes. We defined the region −3000 to +1000 from the gene transcription start site as the mapping scale. If a CpG island site was located within this scale, it was defined as mapped to the corresponding gene.

### Bisulfite-specific PCR sequencing experiments

One microgram of DNA of cancer and adjacent tissue was bisulfite-modified using a QIAGEN epitect kit. Bisulfite-specific PCR (BSP) primers were designed using Methyl Primer Express v1.0 (ABI). For BSP sequencing, target fragments were amplified according to the following sequence: 96 °C for 3 min followed by a touchdown program with 10 cycles (94 °C for 25 s, (Tm + 3) °C for 25 s, and 72 °C for 30 s), and 40 cycles (94 °C for 25 s, (Tm-2) °C for 25 s, and 72 °C for 30 s). Tm was calculated using Primer Premier 5.0.

PCR amplicons were subcloned into the pMD-18T vector (TaKaRa), and 16 clones were picked and tested using the vector primers (pMD18-124F: 5′-CCAGGGTTTTCCCAGTCACG-3′; pMD18-124R: 5′-AAACAGCTATGACCATGATTACGAA-3′) to screen the clones with insert fragment of the right size. Twelve clones were sequenced to evaluate the methylation distribution of the cancer tissue and adjacent tissue. We selected *ANKRD45, APC, CDX1, HOXD3* and TUBB6 genes and used the bisulfite PCR method to validate the array data.

### RT-PCR

Total RNA was extracted using TRIzol (Invitrogen) and reverse transcribed using an iScript cDNA synthesis kit (BIO-RAD, USA). Real-time PCR was performed on an ABI7900 (ABI) instrument using the Taqman Universal PCR Master Mix (ABI, USA) according to the manufacturer’s protocol. The data were normalized to the reference gene *GAPDH*. The primer sequences were Gapdh-F: 5′-TGACTTCAACAGCGACACCCA-3′, Gapdh-R: 5′-CACCCTGTTGCTGTAGCCAAA-3′; *ANKRD45*-F: 5′-GCTCGAGATGTTGCTGCTAGATATT-3′, *ANKRD45*-R: 5′-TTTTTTCAGAGTCAGCCTTGCA-3′; and *HOXD3*-F: 5′-GGCCAGCGTGGACTACAGTT-3′, *HOXD3*-R: 5′-GAGAGATCTGTGTAGGTGGGATGA-3′.

The relative expression levels of *ANKRD45* and *HOXD3* in HCC cancer and adjacent tissue were calculated using the 2^−ΔΔCT^ method^38^.

## Additional Information

**How to cite this article:** Yang, Y. *et al*. A New Approach to Evaluating Aberrant DNA Methylation Profiles in Hepatocellular Carcinoma as Potential Biomarkers. *Sci. Rep.* **7**, 46533; doi: 10.1038/srep46533 (2017).

**Publisher's note:** Springer Nature remains neutral with regard to jurisdictional claims in published maps and institutional affiliations.

## Supplementary Material

Supplemental Files

## Figures and Tables

**Table 1:** The list of genes that had methylated peaks in more than 3 samples in our results.

gene symbol	other name	location	Correlated samples
PGR	PR	11q22	23
MYC	c-Myc	8q24.21	22
APC	DP2	5q21	21
ESR1	ER	6q25.1	21
GATA6		18q11.1-q11.2	18
CDKN1B	p27	12p13.1-p12	16
CDKN1C	p57	11p15.5	16
SFRP2	SARP1	4q31.3	16
MYOD1	MYOD	11p15.4	14
RARB	RARβ2, Hap	3p24	14
MGMT		10q26	12
PRLR	hPRLrI	5p13-5p12	11
CDKN2B	p15	9p21	10

## References

[b1] Gardiner-Garden M. & Frommer M. CpG islands in vertebrate genomes. Journal of molecular biology 196(2), 261–282 (1987). 3656447 10.1016/0022-2836(87)90689-9

[b2] Ioshikhes I. P. & Zhang M. Q. Large-scale human promoter mapping using CpG islands. Nature genetics 26(1), 61–63 (2000). 10973249 10.1038/79189

[b3] Caiafa P. & Zampieri M. DNA methylation and chromatin structure: the puzzling CpG islands. J Cell Biochem 94(2), 257–265 (2005). 15546139 10.1002/jcb.20325

[b4] Antequera F. Structure, function and evolution of CpG island promoters. Cell Mol Life Sci 60(8), 1647–1658 (2003). 14504655 10.1007/s00018-003-3088-6 PMC11138798

[b5] Illingworth R. . A novel CpG island set identifies tissue-specific methylation at developmental gene loci. PLoS Biol 6(1), e22 (2008). 18232738 10.1371/journal.pbio.0060022 PMC2214817

[b6] Feil R. & Khosla S. Genomic imprinting in mammals: an interplay between chromatin and DNA methylation? Trends Genet 15(11), 431–435 (1999). 10529801 10.1016/s0168-9525(99)01822-3

[b7] Shi H., Wang M. X. & Caldwell C. W. CpG islands: their potential as biomarkers for cancer. Expert Rev Mol Diagn 7(5), 519–531 (2007). 17892361 10.1586/14737159.7.5.519

[b8] Esteller M., Corn P. G., Baylin S. B. & Herman J. G. A gene hypermethylation profile of human cancer. Cancer Res 61(8), 3225–3229 (2001). 11309270

[b9] Feinberg A. P. & Vogelstein B. Hypomethylation distinguishes genes of some human cancers from their normal counterparts. Nature 301(5895), 89–92 (1983). 6185846 10.1038/301089a0

[b10] Jones P. A. & Baylin S. B. The epigenomics of cancer. Cell 128(4), 683–692 (2007). 17320506 10.1016/j.cell.2007.01.029 PMC3894624

[b11] Hansen K. D. . Increased methylation variation in epigenetic domains across cancer types. Nat Genet 43(8), 768–775 (2011). 21706001 10.1038/ng.865 PMC3145050

[b12] Costello J. F. . Aberrant CpG-island methylation has non-random and tumour-type-specific patterns. Nat Genet 24(2), 132–138 (2000). 10655057 10.1038/72785

[b13] Feinberg A. P., Ohlsson R. & Henikoff S. The epigenetic progenitor origin of human cancer. Nat Rev Genet 7(1), 21–33 (2006). 16369569 10.1038/nrg1748

[b14] Hendrich B. & Bird A. Identification and characterization of a family of mammalian methyl-CpG binding proteins. Mol Cell Biol 18(11), 6538–6547 (1998). 9774669 10.1128/mcb.18.11.6538 PMC109239

[b15] Ng H. H. . MBD2 is a transcriptional repressor belonging to the MeCP1 histone deacetylase complex. Nat Genet 23(1), 58–61 (1999). 10471499 10.1038/12659

[b16] Yildirim O. . Mbd3/NURD complex regulates expression of 5-hydroxymethylcytosine marked genes in embryonic stem cells. Cell 147(7), 1498–1510 (2011). 22196727 10.1016/j.cell.2011.11.054 PMC3252821

[b17] Baubec T., Ivanek R., Lienert F. & Schubeler D. Methylation-dependent and -independent genomic targeting principles of the MBD protein family. Cell 153(2), 480–492 (2013). 23582333 10.1016/j.cell.2013.03.011

[b18] Jiang C. L., Jin S. G. & Pfeifer G. P. MBD3L1 is a transcriptional repressor that interacts with methyl-CpG-binding protein 2 (MBD2) and components of the NuRD complex. J Biol Chem 279(50), 52456–52464 (2004). 15456747 10.1074/jbc.M409149200

[b19] Fraga M. F. . The affinity of different MBD proteins for a specific methylated locus depends on their intrinsic binding properties. Nucleic Acids Res 31(6), 1765–1774 (2003). 12626718 10.1093/nar/gkg249 PMC152853

[b20] Ballestar E. . Methyl-CpG binding proteins identify novel sites of epigenetic inactivation in human cancer. EMBO J 22(23), 6335–6345 (2003). 14633992 10.1093/emboj/cdg604 PMC291845

[b21] Lopez-Serra L. . A profile of methyl-CpG binding domain protein occupancy of hypermethylated promoter CpG islands of tumor suppressor genes in human cancer. Cancer Res 66(17), 8342–8346 (2006). 16951140 10.1158/0008-5472.CAN-06-1932

[b22] Rauch T., Li H., Wu X. & Pfeifer G. P. MIRA-assisted microarray analysis, a new technology for the determination of DNA methylation patterns, identifies frequent methylation of homeodomain-containing genes in lung cancer cells. Cancer Res 66(16), 7939–7947 (2006). 16912168 10.1158/0008-5472.CAN-06-1888

[b23] Wood K. H. & Zhou Z. Emerging Molecular and Biological Functions of MBD2, a Reader of DNA Methylation. Front Genet 7, 93 (2016). 27303433 10.3389/fgene.2016.00093 PMC4880565

[b24] Dahl C. & Guldberg P. DNA methylation analysis techniques. Biogerontology 4(4), 233–250 (2003). 14501188 10.1023/a:1025103319328

[b25] Lister R. . Highly integrated single-base resolution maps of the epigenome in Arabidopsis. Cell 133(3), 523–536 (2008). 18423832 10.1016/j.cell.2008.03.029 PMC2723732

[b26] Lister R. . Human DNA methylomes at base resolution show widespread epigenomic differences. Nature 462(7271), 315–322 (2009). 19829295 10.1038/nature08514 PMC2857523

[b27] Bock C. . Quantitative comparison of genome-wide DNA methylation mapping technologies. Nat Biotechnol 28(10), 1106–1114 (2010). 20852634 10.1038/nbt.1681 PMC3066564

[b28] Tian X., Sun D., Zhao S., Xiong H. & Fang J. Screening of potential diagnostic markers and therapeutic targets against colorectal cancer. Onco Targets Ther 8, 1691–1699 (2015). 26185457 10.2147/OTT.S81621 PMC4501159

[b29] Scacheri P. C., Crawford G. E. & Davis S. Statistics for ChIP-chip and DNase hypersensitivity experiments on NimbleGen arrays. Methods Enzymol 411, 270–282 (2006). 16939795 10.1016/S0076-6879(06)11014-9

[b30] Esteller M. CpG island hypermethylation and tumor suppressor genes: a booming present, a brighter future. Oncogene 21(35), 5427–5440 (2002). 12154405 10.1038/sj.onc.1205600

[b31] Tischoff I. & Tannapfe A. DNA methylation in hepatocellular carcinoma. World J Gastroenterol 14(11), 1741–1748 (2008). 18350605 10.3748/wjg.14.1741 PMC2695914

[b32] Issa J. P. CpG island methylator phenotype in cancer. Nat Rev Cancer 4(12), 988–993 (2004). 15573120 10.1038/nrc1507

[b33] Hellman A. & Chess A. Gene body-specific methylation on the active X chromosome. Science 315(5815), 1141–1143 (2007). 17322062 10.1126/science.1136352

[b34] Down T. A. . A Bayesian deconvolution strategy for immunoprecipitation-based DNA methylome analysis. Nat Biotechnol 26(7), 779–785 (2008). 18612301 10.1038/nbt1414 PMC2644410

[b35] Hua Y. J., Tu K., Tang Z. Y., Li Y. X. & Xiao H. S. Comparison of normalization methods with microRNA microarray. Genomics 92(2), 122–128 (2008). 18514480 10.1016/j.ygeno.2008.04.002

[b36] Smyth G. K. & Speed T. Normalization of cDNA microarray data. Methods 31(4), 265–273 (2003). 14597310 10.1016/s1046-2023(03)00155-5

[b37] Wang Y. W. . ITPKA Gene Body Methylation Regulates Gene Expression and Serves as an Early Diagnostic Marker in Lung and Other Cancers. J Thorac Oncol 11(9), 1469–1481 (2016). 27234602 10.1016/j.jtho.2016.05.010 PMC5555593

[b38] Livak K. J. & Schmittgen T. D. Analysis of relative gene expression data using real-time quantitative PCR and the 2(-Delta Delta C(T)) Method. Methods 25(4), 402–408 (2001). 11846609 10.1006/meth.2001.1262

