# Supplementary material for: A New Approach to Evaluating Aberrant DNA Methylation Profiles in Hepatocellular Carcinoma as Potential Biomarkers
Source: Sci Rep. 2017 Apr 18;7:46533. doi: 10.1038/srep46533 (PMC5394454; doi:10.1038/srep46533)
Supplement: Supplemental Files [file srep46533-s1.doc]

# A New Approach to Evaluating Aberrant DNA Methylation Profiles in Hepatocellular Carcinoma as Potential Biomarkers

Yuan Yang^1,*^, Linghao Zhao^1,*^, Bo Huang ^2,*^, Guojun Hou^1^, Beibei Zhou^3^, Jin Qian^3^, Shengxian Yuan^1^, Huasheng Xiao^3^, Minghui Li^3,$^, Weiping Zhou^1,$^

^1^The Third Department of Hepatic Surgery, Eastern Hepatobiliary Surgery Hospital, Second Military Medical University, Shanghai, China;

^2^ Suzhou Municipal Hospital, Jiangsu Province, China;

^3^ Shanghai Biotechnology Corporation, Shanghai, China;

*These authers contributed equally to this work.

^$^Corresponding auther:

Weiping Zhou: [ehphwp@126.com](mailto:ehphwp@126.com), Eastern Hepatobiliary Surgery Hospital, 225 Changhai Road, 200438, Shanghai, P.R.China. Tel: (+86) 021-81875521; Fax: (+86) 021-81875529.

Minghui Li: minghui_li@shbiochip.com , Tel:(+86) 021-51320288;

Supplementary Table1: Primer used in this paper.

|  | primer name | sequence |
| --- | --- | --- |
| BSP Primer | APC-270BF: | 5' GGGTTAGGGTTAGGTAGGTTGTG 3' |
|  | APC-270BR: | 5' CCATAATAACTCCAACACCTACCC 3' |
|  | CDX1-326BF： | 5' TTTGTAAAATGGGGTTGTAGGGT 3' |
|  | CDX1-326BR： | 5' CCCCACCCAAACCTTTTATAAC 3' |
|  | ANKRD-261BF: | 5' AGGGGAGTGGGTTTTAATTTTAG |
|  | ANKRD-261BR: | 5' CCAAAAACAACCCTAAACACAC |
|  | HOXD3 260BFr: | 5' TAAGGTTTATGGTGGTGGTTGTTT 3' |
|  | HOXD3 260BRr: | 5' CCACTCAACAACTACCTACCACAA 3' |
|  | NHLRC1 371BF: | 5' GTTGAGTTTAGGAGTTTTATGAGGT 3' |
|  | NHLRC1 371BR: | 5' ACCCCRTAACCATAACTATAACC 3' |
|  | CD55-294BF: | 5' TTTGATAGATTTTTTAGTAGAGGG 3' |
|  | CD55-294BF: | 5' TCTAAAACTCACTCTCTCCACTA 3' |
|  | RIMBP2 226BF: | 5' GGTTAGGGGAGGAAAGAAAATATA 3' |
|  | RIMBP2 226BR: | 5' TCTCACCTACCCTCTCCTTAACTA 3' |
| Spike-in Real Time Primer | Ara30-376F: | 5' CGAAAGCAACAGAAGCAAAAC 3' |
|  | Ara30-376R: | 5' TACGGATGGAGCCACGAA 3' |
|  | Ara30-242F: | 5' ATAACCGTGGCAACATCGTC 3' |
|  | Ara30-242R: | 5' AAGGCGGAGAAGTCTGAGGA 3' |
|  | Ara37-633F: | 5' GGTTGATGGACCCACTTGTTT 3' |
|  | Ara37-633R: | 5' CCGATTGTATTCCCTTTATTGC 3' |
|  | Ara37-249F: | 5' GGAGGTCGTAGCAGTGGTAGTG 3' |
|  | Ara37-249R: | 5' GGATCCGGTTCCCATGTTATA 3' |
| Spike-in Primer | Araisland6-517F: | 5' GTAAAGGGAGAAAGCACCA 3' |
|  | Araisland6-517R: | 5' ACGATGACCGAATCAAGC 3' |
|  | Ara14-1193F: | 5' CGCCATTATCCTTCACCC 3' |
|  | Ara14-1193R: | 5' CTTGCCACCACTCCCTGT 3' |
|  | araisland2-666F: | 5' GCGGAATGGAGAAGACAG 3' |
|  | araisland2-666R: | 5' GGATTTCACGAGGGTTGT 3' |

Supplementary Table 2: 66 Tumor suppressor genes( TSGs) that have been reported frequently.

| gene symbol | other name | location | description |
| --- | --- | --- | --- |
| ABO | A3GALNT, A3GALT1, GTB, NAGAT | 9q34.1-q34.2 | ABO blood group (transferase A, alpha 1-3-N-acetylgalactosaminyltransferase; transferase B, alpha 1-3-galactosyltransferase) |
| APC | DP2 | 5q21 | adenomatous polyposis coli，BTPS2, DP2, DP2.5, DP3, GS |
| AR | TFM | Xq11 | RP11-383C12.1, AIS, DHTR, HUMARA, HYSP1, KD, NR3C4, SBMA, SMAX1, TFM |
| BRCA1 |  | 17q21 | breast cancer 1, early onset |
| CASP8 | ALPS2B | 2q33-q34 | caspase 8, apoptosis-related cysteine peptidase; FADD-homologous ICE/CED-3-like protease |
| CDH1 | E-cadherin | 16q22.1 | cadherin 1, type 1, E-cadherin(epithelial)，calcium-dependent adhesion protein |
| CDH13 | H-cadherin | 16q24.2-q24.3 | cadherin 13, H-cadherin (heart) |
| CDH15 | M-cadherin | 16q24.3 | cadherin 15, type 1, M-cadherin (myotubule) |
| CDKN1A | p21 | 6p21.2 | cyclin-dependent kinase inhibitor 1A; melanoma differentiation associated protein 6 |
| CDKN1B | p27 | 12p13.1-p12 | cyclin-dependent kinase inhibitor 1B |
| CDKN1C | p57 | 11p15.5 | cyclin-dependent kinase inhibitor 1C |
| CDKN2A | p16 | 9p21 | cyclin-dependent kinase inhibitor 2A |
| CDKN2B | p15 | 9p21 | cyclin-dependent kinase inhibitor 2B; |
| COX5A | COX | 15q24.1 | cytochrome c oxidase subunit Va |
| DAPK1 | DAPK | 9q34.1 | death-associated protein kinase 1 |
| DAPK2 | DRP-1, MGC119312 | 15q22.31 | death-associated protein kinase |
| DAPK3 | FLJ36473, ZIP, ZIPK | 19p13.3 | ZIP kinase isoform |
| DBC1 | DBCCR1 | 9q32-q33 | deleted in bladder cancer 1 |
| ESR1 | ER | 6q25.1 | estrogen receptor alpha; estrogen receptor alpha delta 4,5,7 isoform |
| FAT1 | FAT | 4q34-35 | CDHF7, FAT, ME5, hFat1 |
| FHIT | AP3Aase, FRA3B | 3p14.2 | fragile histidine triad gene |
| GALR2 | GALNR2 | 17q25.3 | galanin receptor 2 |
| GATA4 | MGC126629 | 8p23-p22 | GATA binding protein 4 |
| GATA5 | bB379O24.1 | 20q13 | GATA binding protein 5 |
| GATA6 | no | 18q11.1-q11.2 | GATA-binding protein 6; transcription factor GATA-6 |
| GSTP1 |  | 11q13 | fatty acid ethyl ester synthase III; glutathione transferase |
| HIC1 | hic-1 | 17p13.3 | ZBTB29, hic-1 |
| IRF7 | IRF7A | 11p15.5 | interferon regulatory factor 7 |
| MGMT |  | 10q26 | methylguanine-DNA methyltransferase |
| MLH1 | hMLH1 | 3p21.3 | DNA mismatch repair protein Mlh1 |
| MSH2 | hMSH2 | 2p22-p21 | mutS homolog 2, colon cancer, nonpolyposis type 1 |
| MSH3 | hMSH3 | 5q11-q12 | mutS homolog 3 (E. coli) |
| MT1A | MT1 | 16q13 | metallothionein 1A |
| MYC | c-Myc | 8q24.21 | v-myc myelocytomatosis viral oncogene homolog (avian) |
| MYOD1 | MYOD | 11p15.4 | myogenic differentiation 1 ;myoblast determination protein 1，MYF3, MYOD, PUM, bHLHc1 |
| PENK |  | 8q23-q24 | enkephalin A; preproenkephalin |
| PGR | PR | 11q22 | progesterone receptor |
| PLXNA2 | OCT | 1q32.2 | plexin A2 |
| PRDM2 | RIZ1 | 1p36 | PR domain containing 2, with ZNF domain |
| PRLR | hPRLrI | 5p13-5p12 | prolactin receptor |
| PTGS2 | COX-2 | 1q25.2-q25.3 | prostaglandin-endoperoxide synthase 2，COX-2, COX2, GRIPGHS, PGG/HS, PGHS-2, PHS-2, hCox-2 |
| PYCARD | TMS1, ASC | 16p12-p11.2 | PYD and CARD domain containing,ASC, CARD5, MGC10332, TMS, TMS-1, TMS1 |
| RARB | RARβ2，Hap | 3p24 | HAP, NR1B2, RRB2 |
| RASSF1 | RASSF1A | 3p21.3 | 123F2, NORE2A, RASSF1A, RDA32, REH3P21，MGC94319 |
| RASSF5 | NORE1A | 1q32 | Maxp1, Nore1，RP11-343H5.1, MGC10823, MGC17344, NORE1A, NORE1B, RAPL, RASSF3 |
| RB1 | Rb | 13q14.2 | OSRC, RB, p105-Rb, pRb, pp110 |
| RP | BLOS3, FLJ26641, FLJ26676, HPS8 | 19q13.32 | biogenesis of lysosome-related organelles complex-1 |
| RUNX3 | FLJ34510 | 1p36 | runt-related transcription factor 3 |
| SALL3 | ZNF796 | 18q23 | sal-like 3 |
| SEMA3B | FLJ34863, LUCA-1, SEMA5, SEMAA, SemA, semaV | 3p21.3 | sema domain, immunoglobulin domain (Ig), short basic domain, secreted, (semaphorin) 3B |
| SFRP1 | SARP2 | 8p12-p11 | secreted frizzled-related protein 1,FRP, FRP-1, FRP1, FrzA, SARP2 |
| SFRP2 | SARP1 | 4q31.3 | secreted frizzled-related protein 2 |
| SFRP4 | FRP-4, FRPHE, MGC26498 | 7p14.1 | secreted frizzled-related protein 4 |
| SFRP5 | SARP3 | 10q24.1 | secreted apoptosis related protein 3 |
| SOCS1 | TIP3 | 16p13.13 | suppressor of cytokine signaling 1，CIS1, CISH1, JAB, SOCS-1, SSI-1, SSI1, TIP3 |
| SOCS3 | SOCS-3 | 17q25 | suppressor of cytokine signaling 3， ATOD4, CIS3, Cish3, MGC71791, SOCS-3, SSI-3, SSI3 |
| SPARC |  | 5q31.3-q32 | secreted protein, acidic, cysteine-rich (osteonectin) |
| STK11 | LKB1 | 19p13 | polarization-related protein LKB1; serine/threonine protein kinase 11 |
| SYK | DKFZp313N1010 | 9q22 | spleen tyrosine kinase，DKFZp313N1010, FLJ25043, FLJ37489 |
| THBS1 | TSP1 | 15q15 | THBS, THBS-1, TSP, TSP1 |
| TIMP3 | SFD | 22q12.3 | TIMP metallopeptidase inhibitor 3 |
| TMEFF2 | HPP1 | 2q32.3 | transmembrane protein with EGF-like and two follistatin-like domains 2,HPP1, TENB2, TPEF, TR |
| TP73 | p73 | 1p36 | p53-like transcription factor |
| VCAN | CSPG2 | 5q14.3 | versican; Wagner syndrome (erosive vitreoretinopathy) |
| VHL | VHL1 | 3p25 | HRCA1, RCA1, VHL1 |
| ZMYND10 | BLU | 3p21.3 | zinc finger, MYND-type containing 10 |

Supplementary Table 3: Demographic and clinicopathologic characteristics of 58 HCC patients

| Variables | Mean ± SD / n (%) |
| --- | --- |
| **Age**, years | 53.0 ±9.3 |
| **Sex** |  |
| Male | 43(74.1%) |
| Female | 15(25.9%) |
| **HBsAg status** |  |
| positive | 58 (100.0%) |
| **TBIL, µmol/L** | 19.9 ±10.2 |
| **ALB, g/L** | 41.0 ±4.5 |
| **PT, seconds** | 12.5 ±1.1 |
| **AFP, µg/L** | 205.9 ±411.2 |
| **Tumor diameter, cm** | 5.3 ±3.2 |
| **Edmonson-Steiner classification** |  |
| I- II | 36 (62.1%) |
| III- IV | 22 (37.9%) |
| **Microvascular invasion** |  |
| Absence | 20 (34.5%) |
| Presence | 38 (65.5%) |
| **Satellite nodules** |  |
| No | 46 (79.3%) |
| Yes | 12 (20.7%) |
| **Tumor Capsule** |  |
| Complete | 24 (41.4%) |
| Incomplete | 26 (44.8%) |
| No | 8 (13.8%) |
| **Cirrhosis** |  |
| No | 8 (13.8%) |
| Yes | 50 (86.2%) |
| **Child-Pugh grade** |  |
| A | 56 (96.6%) |
| B | 2 (3.4%) |
|  |  |
